# Recent global decrease in the inner-core rain rate of tropical cyclones

**DOI:** 10.1038/s41467-021-22304-y

**Published:** 2021-03-29

**Authors:** Shifei Tu, Jianjun Xu, Johnny C. L. Chan, Kian Huang, Feng Xu, Long S. Chiu

**Affiliations:** 1 South China Sea Institute of Marine Meteorology & College of Ocean and Meteorology, Guangdong Ocean University, Zhanjiang, China; 2 Shenzhen Institute of Guangdong Ocean University, Shenzhen, China; 3 School of Energy and Environment, City University of Hong Kong, Hong Kong, China; 4 Department of Atmospheric, Oceanic and Earth Sciences, George Mason University, Fairfax, VA USA

**Keywords:** Atmospheric science, Climate change

## Abstract

Heavy rainfall is one of the major aspects of tropical cyclones (TC) and can cause substantial damages. Here, we show, based on satellite observational rainfall data and numerical model results, that between 1999 and 2018, the rain rate in the outer region of TCs has been increasing, but it has decreased significantly in the inner-core. Globally, the TC rain rate has increased by 8 ± 4% during this period, which is mainly contributed by an increase in rain rate in the TC outer region due to increasing water vapor availability in the atmosphere with rising surface temperature. On the other hand, the rain rate in the inner-core of TCs has decreased by 24 ± 3% during the same period. The decreasing trend in the inner-core rain rate likely results mainly from an increase in atmospheric stability.

## Abstract (web summary)

How the rainfall intensity of tropical cyclones changes with climate change is not well known. Here, the authors show that while the rain rate in the outer region of TCs is clearly increasing between 1999 and 2018, it decreases significantly in the inner-core of TCs during 1999-2018.

## Introduction

Heavy rainfall from tropical cyclones (TCs) has always been an important research topic^1–4^ because of its impacts such as floods, mudflows and landslides, especially over the coastal areas. TC rain rates have been shown to be a function of TC intensity, with greater rates linked to stronger TCs^2^. Some previous studies^5–7^ have suggested that TC intensity could increase in a warming climate, which implies a possible increase in TC rain rate. The loss of life and property along coastal areas due to TC activity may therefore increase^8^.

Traditional studies on TC rainfall are usually based on rainfall data from rain gauges of land stations^9,10^. With the rapid development of satellite remote sensing technology, the data gaps over the ocean and other difficult-to-observe areas on the earth are largely filled, which has helped to increase our understanding of possible changes in TC rainfall. For example, using the Tropical Rainfall Measuring Mission (TRMM) satellite data and global atmospheric model simulations, Lin et al.^3^ suggested that the rainfall area of a TC is controlled by the relative sea surface temperature (SST), and the precipitation rate increases with absolute SST. The trend variations of TC rainfall in different basins are basically consistent with the large-scale SST and vertical wind shear^11^. In addition, TC-related rainfall shows an increasing trend in many regions^4^, such as North America, East Africa, Southeast Asia and Australia.

Some climate model studies^12,13^ pointed out that the rain rate of TCs may increase by about 3% to 37% by the end of the twenty-first century under global warming. The simulation results^12,14^ further showed different increasing trends within different radii of a TC. For example, Knutson et al.^13^ found that the projected magnitude of rainfall rate is +20% within 100 km region from the TC center. Some studies pointed out that TC rain rate increases tend to be the largest near the TC center, but smaller further from the TC center^4,8,15,16^.

In this work, we use satellite observational rainfall data and a numerical model to investigate the changes of TC rain rate during the period 1999–2018. The results show that while the rain rate in the outer region of TCs is clearly increasing, it decreases significantly in the inner-core during these two decades.

## Results

### Decrease in TC inner-core rain rate

**Fig. 1:**
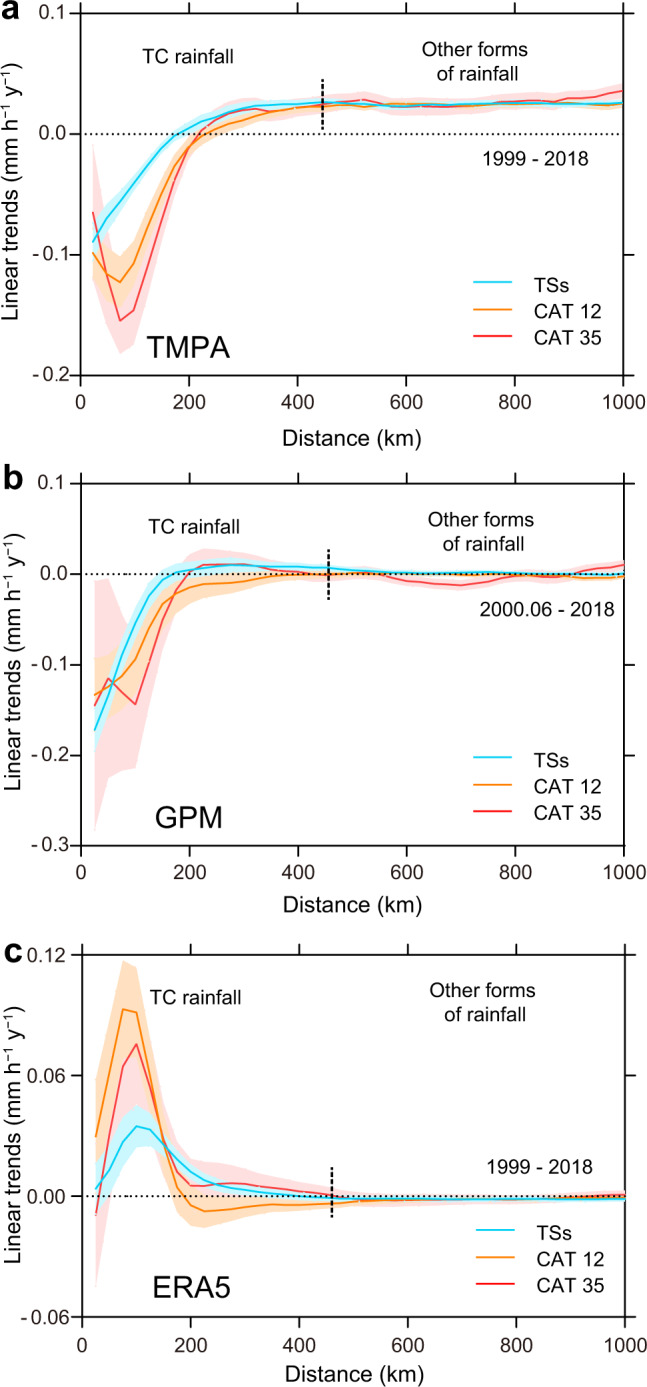
Radial distribution of linear trends (mm h^−1^ y^−1^) of tropical cyclone (TC) rain rate based on different datasets. **a** The Tropical Rainfall Measuring Mission (TRMM) Multi-Satellite Precipitation Analysis (TMPA), **b** Global Precipitation Measurement (GPM), and **c** ERA5 dataset. Shaded areas indicate the standard error of linear trends of the TC rain rate. Blue: tropical storms (TSs), orange: categories 1–2 (CAT12), and red: categories 3–5 (CAT35). The vertical dotted line in each panel indicates the estimated boundary (~450 km for a global scale) of TC rainfall. All the linear trends here consider the rainy pixels only. The area-average results (considering all pixels including rainy and non-rain) are shown in Supplementary Fig. 1.

Most of these previous studies were based on model simulations. However, TC-related rainfall has not been systematically examined using multiple observation platforms or on a global basis. In this study, we therefore investigate the rain rate associated with TCs in all ocean basins using the TMPA (TRMM Multi-satellite Precipitation Analysis) dataset (see “Methods” and Supplementary Table 1 for details). The linear trends of the global TC rain rate (rainy pixels only; results using all pixels are shown in Supplementary Fig. 1a) from TMPA data clearly show a negative trend near the TC center for all TC intensities (Fig. 1a). Furthermore, the decreasing trend is larger for the more intense TCs. Breaking up the dataset into the first and last 5 years also shows that the TC rain rate during the last 5 years (2014–2018) is smaller in the inner-core but greater in the outer region than that during the first 5 years (1999–2018) (Supplementary Fig. 3a). To ascertain that these results are not data dependent, the GPM (Global Precipitation Measurement) data with a shorter period (Supplementary Table 1) are also examined. Although the radial gradient of the linear trends of these two satellite datasets are somewhat different, the GPM dataset also gives a negative trend near the TC center (Fig. 1b and Supplementary Fig. 1b). Thus, it can be concluded that the rain rate in the inner-core of a TC has decreased significantly in recent years.

Note, however, that the results from the global reanalysis (ERA5) data show a positive trend in the rain rate of TC in the inner-core (Fig. 1c, Supplementary Fig. 1c and 3e–f). Possible reasons for this discrepancy between observation and reanalysis will be discussed later in this paper.

### Quantitative analysis of TC rain rate

To quantify the trends in different regions of the TC, and for each of the TC intensity categories, we use the TMPA data to separate the inner-core and outer region of the TC by identifying the location of the maximum gradient (see “Methods” and Supplementary Table 2) on the radial curves of rainfall rate. The inner-core of the TC is then defined as the area from the TC center to this location and the outer region from this location to the boundary (Supplementary Table 2) of the TC rainfall (see “Methods” for details).

**Fig. 2:**
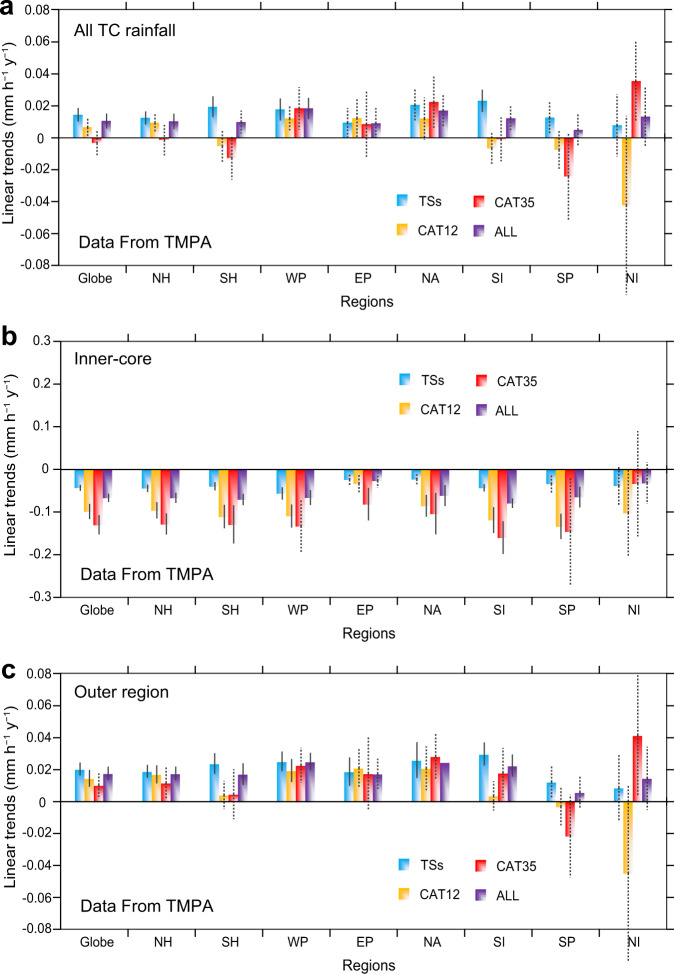
Linear trends (mm h^–1^ y^–1^) of tropical cyclone (TC) rain rate based on the Tropical Rainfall Measuring Mission (TRMM) Multi-Satellite Precipitation Analysis (TMPA) dataset in various ocean basins. **a** All TC rainfall, **b** Inner-core of TC, **c** Outer region of TC. Globe: Globally-averaged, NH: North Hemisphere, SH: South Hemisphere, WP: Western North Pacific, EP: Eastern North Pacific, NA: North Atlantic, SI: South Indian Ocean, SP: South Pacific, NI: North Indian Ocean. The colors are for different TC intensity categories; tropical storms (blue), category 1–2 (orange), category 3–5 (red), and all three types of TCs (purple). The vertical lines indicate the standard error of the linear trends (solid significant at 95%, and dashed insignificant).

Overall, the “all TC” rain rate (integrating the inner-core and outer region of TC) has increased by 8 ± 4% in the last two decades (Fig. 2a and Supplementary Table 3), with an increasing rate of 0.11 ± 0.05 mm h^–1^ per decade. These increasing trends are consistent with most of the climate model results. Note, however, that this increasing trend is only significant in some ocean basins and mostly for weak TCs. More importantly, the results (Fig. 2b) support that, globally, the rain rate over the TC inner-core decreases at a rate of –0.67 ± 0.10 mm h^–1^ per decade. This downward trend is statistically significant for all TC intensity categories and in almost all ocean basins. The magnitude of the changes in the rain rates during 1999~2018 are quite significant (Supplementary Table 3). The decreases are –24 ± 3%, –23 ± 4% and –26 ± 5% globally, and in the Northern and Southern Hemispheres, respectively. These trends of the TC rain rate also vary in different ocean basins. Southern Indian Ocean has shown the largest reduction of –29 ± 4% rate, while the decreases in the western North Pacific and North Atlantic TC rain rates are –21 ± 6% and –22 ± 9%, respectively. Among the three categories of TC, the largest decreasing trend is found in category 3–5 TCs (CAT35), with a decreasing rate of 29% in both Hemispheres.

However, in the outer region of the TC, the rain rate tends to increase over most of the ocean basins, although not all the increases are statistically significant (Fig. 2c). The trend in the Southern Hemisphere is generally greater than that in the Northern Hemisphere (Supplementary Table 3). Percentage-wise, western North Pacific and South Indian Oceans have the largest increase, being 23 ± 5% and 24 ± 7%, respectively. Contrary to the inner-core, changes in the rain rate in the outer region are not statistically significant for CAT35 in most basins, but those for tropical storms (TSs) are.

### Possible contributing factors

**Fig. 3:**
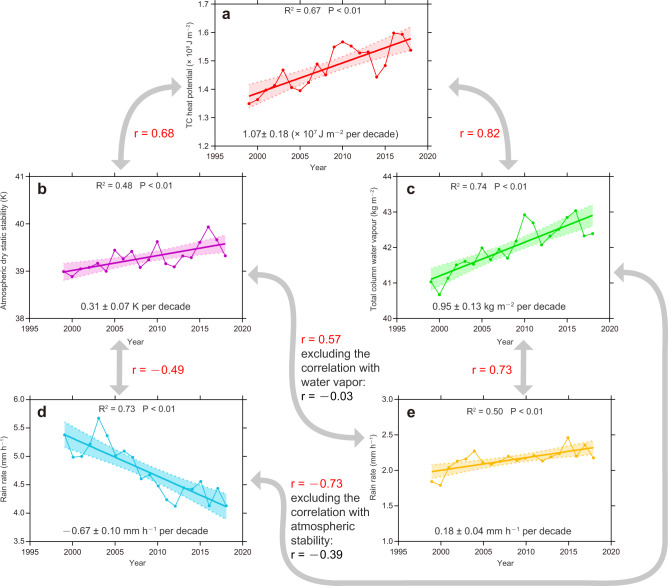
Time series of the different parameters. **a** Tropical cyclone (TC) heat potential (TCHP), unit: ×10^8^ J m^−2^. **b** Atmospheric stability, unit: K. **c** Total column water vapor, unit: kg m^−2^. Moreover, the annual global average rain rate (unit: mm h^–1^) over, the **d** Inner- core, and **e** Outer region of TCs, respectively. The time series in **a**, **b**, and **c** are derived from an average of six ocean basins during the TC peak seasons (Western North Pacific: 120°E–180°,5–30°N, May–December; Eastern North Pacific: 120–90°W,5–30°N, June–October; North Atlantic: 90–20°W,5–30°N, June–October; South Indian Ocean: 50–115°E,5–30°S, November–April; South Pacific: 155°E–180°,5–30°S, December–April; North Indian Ocean: 55–90°E,5–30°N, April–May and September–November). The shading area in all five panels indicate the two-sided 95% confidence levels of the trends, and the bold lines represent the linear regression. The *r* is the correlation coefficients of the two parameters indicated by the gray arrows, red significant and black insignificant. The rain rates in **d** & **e** are constructed using the Tropical Rainfall Measuring Mission (TRMM) Multi-Satellite Precipitation Analysis (TMPA) dataset. All of the linear trends are significant at 95% confidence level.

To understand why the rain rate around the inner-core decreases but increases in the outer region of a TC, two parameters, water vapor and atmospheric stability, that are closely related to rainfall, are examined. As both parameters are related to the heat energy from the ocean, the TC heat potential (TCHP) (see “Methods” for the definition) is analyzed first. The TCHP series (Fig. 3a) shows an increase of 1.07 ± 0.18 × 10^7^ J m^–2^ per decade, which is consistent with the result that the heat content in the upper ocean has an increasing trend in recent years^17^. Under this climate background, the atmospheric stability between 300 and 900 hPa has also increased significantly (Fig. 3b), consistent with the conclusion of Sharmila and Walsh^18^. In addition, there is an increase in the atmospheric moisture content represented by the total column water vapor (Fig. 3c), which has been used as an explanation for more extreme precipitation events in many regions^19,20^.

Previous studies have pointed out that the causes of rainfall in the inner-core and outer regions of a TC differ because of different processes. Strong rising motion in the inner-core leads to more convective rain, while stratiform rain mainly forms in the outer region due to weaker vertical motion^21^. An increase in atmospheric stability (Fig. 3b) would tend to suppress the rising motion, and hence a decreasing rain rate (Fig. 3d). The correlation coefficient between these two-time series is –0.49, which is statistically significant at 95%. On the other hand, water vapor content in the atmosphere increases with rising surface temperature (Fig. 3c). This increase provides additional water vapor to enhance the intensity of rain rate in the TC outer region (Fig. 3e), with the correlation coefficient between the two-time series being 0.73, significant at the 99% confidence level. This feature is basically consistent with the increasing global precipitation intensity^19,20^. As this increase is in the outer region and mainly associated with stratiform rain, it is not related to the TC intensity and hence such an increase is insignificant for more intense TCs (Fig. 2c and Supplementary Table 3).

An increase in water vapor should lead to higher rain rate^22,23^. However, the correlation between inner-core rain rate and water vapor (i.e., between Fig. 3c, d) is negative (*r* = –0.73, *P* < 0.01) and therefore likely not causal. This is supported by the fact that when we remove the correlation with atmospheric stability, the correlation between inner-core rain rate and water vapor is only –0.39, which is insignificant. In other words, the decrease of the rain rate of the TC inner-core is mainly affected by atmospheric stability. Similarly, no significant relationship exists between the increased atmospheric stability and the rain rate of the outer region of TC (i.e. between Fig. 3b, e) when the correlation with water vapor is removed (*r* changing from 0.57 to –0.03).

Clearly, convective rain can occur in both the inner-core and the outer region of TC (such as secondary circulation, and outer spiral rainbands, etc.); and stratiform rain may also be present in the TC inner-core. To understand further the correlation between the two parameters (atmospheric stability and total column water vapor) and rain rate, we follow the results of Liu et al.^24^ and divide the TC rain rates into stratiform (≤4 mm h^–1^) and convective (≥5 mm h^–1^) rain in both the inner-core and outer region of TC (Note: stratiform and convective are mixed in the range of 4–5 mm h^–1^, sample size <5%, so it is not included.). The results show that convective rain occupies a larger proportion (~78%) in the inner-core of TC, while stratiform rain covers more than 84% of the rainy area in the outer region of TC.

**Fig. 4:**
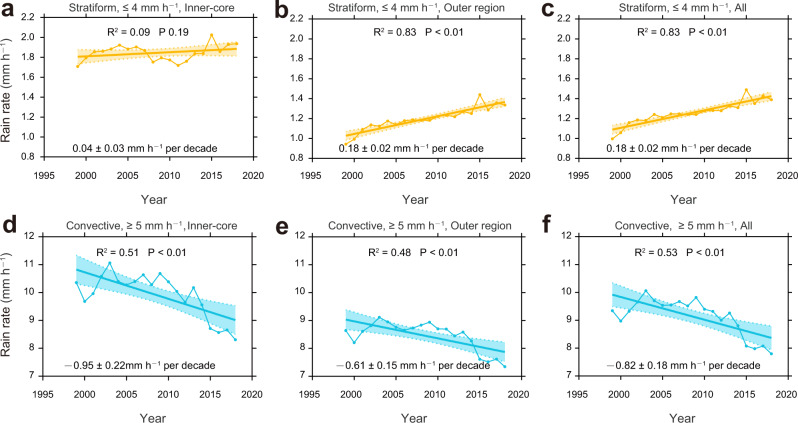
Time series of stratiform and convective rain rate of tropical cyclone (TC). **a**–**c** Stratiform rain rate, **d**–**f** convective rain rate, unit: mm h^−1^. **a**, **d** Inner-core of TC. **b**, **e** Outer region of TC. **c**, **f** Combined inner-core and outer region of TC. The shading area in all panels indicate the two-sided 95% confidence levels of the trends, and the bold lines represent the linear regression. All of the linear trends expect **a** are significant at 95% confidence level. Time series are constructed from the Tropical Rainfall Measuring Mission (TRMM) Multi-Satellite Precipitation Analysis (TMPA) dataset.

Further, in both the inner-core and outer region of TC, the average convective rain rates show a significant weakening trend, being –0.95 mm h^–1^ per decade (*P* < 0.01, Fig. 4d), and –0.61 mm h^–1^ per decade (*P* < 0.01, Fig. 4e), respectively. On the other hand, the increase in the stratiform rain rate is statistically significant only in the outer region (*P* < 0.01, Fig. 4b), but insignificant in the inner-core (*P* = 0.19, Fig. 4a) of the TC. For the entire TC (including inner-core and outer region), the changes of convective and stratiform rain rates are –0.82 mm h^–1^ per decade (*P* < 0.01, Fig. 4f) and 0.18 mm h^–1^ per decade (*P* < 0.01, Fig. 4c), respectively. We also examine the relationship between the atmospheric stability and water vapor with the rain rate of TC (Supplementary Table 4). The results show that atmospheric stability is affecting the convective rain rate in both the inner-core (*r* = –0.50, *P* = 0.02) and outer region (*r* = –0.56, *P* = 0.01) of the TC. However, when the correlation of atmospheric stability is removed, the impact of the rising atmospheric water vapor conditions on convective rain is almost negligible (r changing from –0.45 to –0.05 in the inner-core and –0.48 to –0.05 in the outer region, respectively). The impact of atmospheric water vapor conditions is mainly reflected in the changes of the stratiform rain in the outer region of TC (*r* = 0.83, *P* < 0.01). These results substantiate our earlier discussion that the change in convective rain mainly contributes to the change of rain rate in the inner-core, while the stratiform rain mainly contributes that in the outer region, and hence the relationships of the TC rain rate with atmospheric stability and water vapor content, as shown in Fig. 3.

### Numerical simulations

**Fig. 5:**
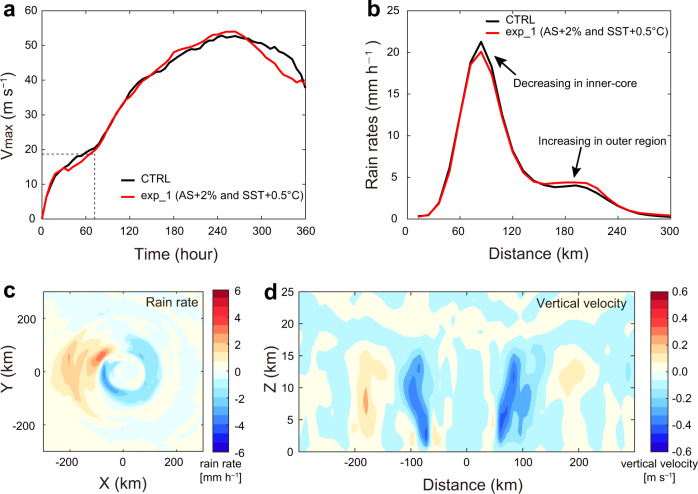
Tropical cyclone (TC) intensity, rain rate, and vertical velocity of TC in the numerical experiments. CTRL (black line): the default Jordan’s sounding and 28 °C sea surface temperature (SST), exp_1 (red line): the atmospheric stability increases by 2% and SST increases by 0.5 °C based on CTRL. **a** The running mean of 5-points of the maximum wind speed (*V*_max_), unit: m s^−1^. **b** The radial rain rate, unit: mm h^−1^. **c** The difference in rain rate (exp_1 – CTRL), unit: mm h^−1^, and **d** The difference in vertical velocity (exp_1 – CTRL) in a vertical east-west cross-section across the TC center, unit: m s^–1^. Details of the experimental design are described in “Methods”.

To investigate whether these proposed mechanisms are indeed plausible, the Weather Research and Forecasting (WRF) model is run using an ideal TC. The control (CTRL) experiment uses the default Jordan’s sounding^25^ and 28 °C SST (the model configuration and physical parameterization schemes described in Methods and Supplementary Table 5). As the atmospheric stability has increased by ~2% during the recent 20 years (Fig. 3b), and the global surface temperature has also increased during this period, the atmospheric stability is increased by 2% and the SST increased to 28.5 °C in exp_1, to make the simulation closer to the observed change. Details of the model experiments are provided in Methods. For both simulations, the maximum wind speed have reached TS intensity after 72 h (Fig. 5a), We, therefore, have calculate the average rain rate and vertical velocity during the 72–360 h integration period. The radial distributions of TC rain rates of these two experiments show that the rain rate of exp_1 decreases in the inner-core but increases in the outer region compared with that in CTRL (Fig. 5b), which are consistent with the statistical results from satellite datasets shown in Supplementary Fig. 3a, c. The spatial pattern of the rain rate difference between CTRL and exp_1 shows a similar result (Fig. 5c). The increase in atmospheric stability leads to a significant weakening of the vertical velocity in the TC eyewall area (60–100 km from the TC center, Fig. 5d), and hence decrease in rain rate in the inner-core (Fig. 5b, c). On the other hand, the increase in SST causes an increase in water vapor content and vertical velocity resulting in an increase in the rain rate of the outer region.

We further calculate the changes in stratiform and convective rain based on the method described earlier. The average stratiform and convective rain of CTRL are 0.76 mm h^–1^ and 12.26 mm h^–1^, while those in exp_1 are 0.78 mm h^–1^ and 11.69 mm h^–1^, respectively, which implies a weakening in convective rain but a strengthening in stratiform rain. These numerical simulation results, therefore, substantiate our earlier hypothesis on the synergistic effect of atmospheric stability and atmospheric water vapor content that leads to a decrease in rainfall rate in the TC inner-core but an increase of rain rate in the outer region of TC.

## Discussion

Despite the consistency between the observational and modeling results, there is still a question of the veracity of the TMPA data, because Chen et al.^26^ pointed out that TMPA 3B42 products generally overestimates TC rain for low rain rate but underestimates TC rain at high rain rate. To reduce the impact of data uncertainty, we have therefore in this study divided TC rainfall into the inner-core with heavier rainfall and the outer region with weaker rainfall, and we use GPM data to verify the results. Furthermore, even if there is an overestimate, it will likely be systematic so that the trends we have identified will still exist.

Note, however, that the trend of TC rain rate from ERA5 (Fig. 1c and Supplementary Fig. 3e–f) is basically contrary to that from satellite observations. One possibility for such a discrepancy is that observational data to be ingested into the reanalyses have to go through quality checks. Rainfall data near the TC center might be considered to be “outliers” and therefore rejected so that the reanalyses are based mostly on the short-term model predictions. However, these predictions have been known to under-represent the TC circulation near the center, and generally under-predict TC intensity^27^. One possible reason for such an under-prediction is that the structure of a TC is not well represented in the model. If the model had a good TC structure, it could have simulated the differences in the rain rate between the inner-core and the outer regions.

The high rain rate in the inner-core of a TC is often accompanied by destructive winds, which poses a huge threat to life and property^28^. While our results suggest a decrease in TC rain rate in the inner-core, other studies have shown a possibility of an increase in the TC intensity^13^. Note, however, that because the rain rate in the outer region is increasing, as well as the total TC rain rate, disaster preparedness efforts must take all these into consideration. For example, a reduction in the inner-core rain rate does not necessarily imply a decrease in the threat from TC rainfall. Indeed, because of the increase in outer region rain rate, coastal areas should be prepared earlier even before a TC is likely to come close to shore.

This study has shown that the rain rate in the inner-core of a TC in almost all ocean basins has a significant decreasing trend due to an increase in atmospheric stability. However, this does not mean that the reduced rain rate is only affected by this factor. In fact, only about 24% of the variance of the inner-core rain rate is explained by the variation in atmospheric stability (Fig. 3). Further analyses and numerical experiments are, therefore, necessary to identify other contributing factors.

The moist adiabatic lapse rate is an important factor affecting the static stability of tropical regions, especially the tropical oceans^29,30^. As a TC contains sufficient water vapor, the changes of water vapor content during the process of rainfall formation in a TC could impact the moist adiabatic lapse rate. Therefore, it is important to understand the relationship between the stability, water vapor, and the moist adiabatic lapse rate, which may require high-resolution observations and model data.

Another important finding from this study is that current reanalysis data tend to overestimate the positive response of TC rain rate to the rising temperature, especially in the inner-core of TC. The possible reasons for the discrepancy in reanalysis datasets have been discussed earlier. As of limited data availability, this study has only investigated the changes in the TC rain rate during the last two decades. It is unclear whether the decrease of the inner-core rain rate (or the convective rain rate) will continue in the future. There is no definite evidence as to how atmospheric stability might change in the future. As the global surface temperature increases, if atmospheric stability is weakened or remains basically unchanged, the TC rain rate may increase with the rising absolute moisture content in the future. However, if atmospheric stability increases with global warming, the inner-core rain rate (or convective rain rate) of the TC may continue to decrease in the future. Therefore, more efforts should be made to improve the ability of the models in simulating or projecting the TC rain rate. In particular, more studies are needed to investigate the relationship between TC rain rate variability and atmospheric stability under global warming.

## Methods

### Data

The historical best-track data of TC are taken from the International Best Track Archive for Climate Stewardship-The World Meteorological Organization (IBTrACS-WMO) v4 dataset^31^, which includes the position, minimum sea-level pressure, and the maximum sustained wind speed of TCs from 1999–2018. The TC-related precipitation data are obtained from TRMM Multi-satellite Precipitation Analysis (TMPA) 3B42 v7^32^, Global Precipitation Measurement (GPM) IMERG Final Precipitation L3 Half Hourly 0.1 degree x 0.1 degree V06^33^. To make a comparison with TC rain rate from the satellite rainfall products, the ERA5^34^ data are also used.

The annual averaged TC heat potential (TCHP) is obtained from IAP Gridded temperature dataset^35^ (v3, 1 degree, monthly, 0–2000 m). The total column water vapor and the atmospheric stability are extracted from the monthly ERA-Interim reanalysis dataset^36^.

The details of all the data using in this work are shown in Supplementary Table 1.

### The TC-related rain rate

The IBTrACS-WMO v4 data has various wind averaging periods in TC conditions from different agencies. Accordingly, we convert the 1-min and 3-min mean wind speeds into 10-min mean wind speeds, based on Harper et al.^37^. This work focuses on the rain rate variability of TC over the globe during 1999–2018. First, we have removed these records that not at 00:00, 03:00, 06:00, 09:00, 12:00, 15:00, 18:00, 21:00 UTC. Then, the non-TC systems (such extratropical, wave, disturbance or others) are also removed. Considering the effects of satellite orbital changes, we select TC activity within 40°S–40°N. All TCs with wind speeds in best-track dataset ≥35 knots, on both land and ocean, are considered in this study. Implementing that selection criterion gives a total of 1499 TCs, with 49887 effective instantaneous observations (Supplementary Table 1) of TC rainfall over the world during the 20 years. This is likely the largest TC rainfall dataset ever assembled.

The rainfall of TC is mainly concentrated in the eyewall. To better reflect the changing characteristics of TC precipitation, we define the average rain rate in each instantaneous TC as:

1 $$R_m = \frac{1}{N}\mathop {\sum }\limits_{i = 1}^N p_i$$

where *R*_*m*_ in Eq. (1) is the mean rain rate of the TC rainfall in each time, and *p*_*i*_ is the rain rate of each spatial grid (pixel), and *N* is the count of pixels in each 25km-ring (i.e. 0–25 km, 25–50 km, 50–75 km, …, 975–1000 km).

As there are differences in the TC classification definitions in different ocean basins, the Saffir-Simpson Hurricane Wind Scale is used to categorize the TCs: TCs with maximum surface wind speed near the center at 35–64 knots as tropical storms (TSs), ≥64 knots as hurricanes (or typhoon). The latter TCs are further classified: wind speeds at 64-96 knots as category 1&2 (CAT12), and the wind speed ≥96 knots as category 3–5 (CAT35).

We obtain the radial curves of the rain rate of 49,887 instantaneous TC observations for all ocean basins according to rainy pixels only. Then, we calculate the linear trends within each radial band for the three TC intensities using each of the rainfall datasets, and the results are shown in Fig. 1. The linear trends of the area-average rain rate of TC are shown in Supplementary Fig. 1. In order to quantify the changes of rain rate in the inner-core and outer regions, we further calculate, using the TMPA data, the average radial rain rate distribution curves (all pixels) for the three TC intensity categories over six ocean basins. Then, we define rain rate >0.5 mm h^–1^ as TC rainfall, to obtain the TC mean rainfall radii of the three categories of TC in all the ocean basins (Supplementary Table 2). To separate the inner-core and outer region of the TC, we identify the position of the maximum gradient (Supplementary Table 2) of the average radial rain rate curve. An example of the positions of the maximum gradient of different rain rate radial curves is shown in Supplementary Fig. 2. Using these positions, we calculate the annually average rain rate (raining pixels only), and then the linear trends shown in Fig. 2.

### TC heat potential

The TCHP represents the heat contained in the upper layer of the 26 °C isotherm of the upper ocean. Based on previous studies^38–40^, it is defined as:

2 $${\mathrm{TCHP}} = c_{\mathrm{p}}\rho {\int}_{D_{26}}^0 {[T\left( z \right) - 26]dz}$$

where *c*_p_ in Eq. (2) is the specific heat capacity at constant pressure, usually taken as 4178 J kg^−1^ °C^−1^; *ρ* the density of sea water, taken as 1026 kg m^−3^ in the upper ocean; *D*_26_ the depth of the 26 °C isotherm, and *T(z)* the in-situ temperature from the ocean analysis data. The annual TCHP series is obtained from the average of six ocean basins during the TC peak seasons (WP: 120°E–180°,5–30°N, May–December; EP: 120–90°W,5–30°N, June–October; NA: 90–20°W,5–30°N, June–October; SI: 50–115°E,5–30°S, November–April; SP: 155°E–180°,5–30°S, December–April; NI: 55–90°E,5–30°N, April–May and September–November; same as below).

### Atmospheric stability

According to Sharmila and Walsh (2018)^18^, the atmospheric stability (AS) is defined as:

3 $${\mathrm{AS}} = \theta _{300} - \theta _{900}$$

where *θ*_300_ and *θ*_900_ in Eq. (3) indicate the potential temperature at 300 and 900 hPa, respectively. The potential temperature *θ* is defined as:

4 $$\theta = T\left(\frac{{P_0}}{P}\right)^{\frac{R}{{C_p}}}$$

where, *T* in Eq. (4) is the absolute temperature (K), *R* the gas constant of air, and *c*_p_ the specific heat capacity at a constant pressure, *P*_*0*_ the reference pressure, usually taken as 1000 hPa, and *P* the atmospheric pressure. In general, *R/c*_p_ = 0.286 for air. The absolute temperature is obtained from the monthly ERA-Interim reanalysis dataset. Same as TCHP, the atmospheric stability is obtained from the average of six ocean basins during the TC peak seasons.

### Total column water vapor

This annual time series is obtained from the average value of six ocean basins during the TC peak seasons (same as TCHP).

### Statistical information

The linear trends in Figs. 1–5 and Supplementary Fig. 1 are estimated using simple linear regression of rain rates of TC. The shaded areas in Fig. 1 and Supplementary Fig. 1 and the vertical lines in Fig. 2 are the standard error (*n* = 20) of linear trends of the TC rain rate. Shaded areas in Figs. 3–4 are the two-sided 95% confidence bounds. The percentages in Supplementary Table 3 are calculated by the difference between the last point and first point of the fitted linear regression line and then divided the first point. In this work, the significance of linear trends and the correlation coefficients are using the two-tailed *t*-test (degree of freedom is 18).

### Model configuration and experiments

- Model configuration
  The Advanced Research WRF (ARW) modeling system (version 4.1.3)^41^ is used to simulate the ideal TC. The ideal model has 300 × 300 grid points with a 12 km horizontal resolution. The model top is 25 km with 25 vertical layers. Jordan’s sounding^25^ is used in the simulation, which is the average state of the tropical atmosphere in summer, and it is very nearly neutral to real convection^42^.
  The initial SST is constant (28 °C). An f-plane is assumed. The lateral boundary conditions are periodic. The initial state is motionless (*u* = *v* = 0) and horizontally homogeneous. The model begins the simulation by specifying the vortex tangential velocity^42^ showing in Eq. (5).
  5 $${v\left( {r,z,0} \right) = \frac{{z_{{\mathrm{sponge}}} - z}}{{z_{{\mathrm{sponge}}}}}\left\langle\left\{ {v_m^2\left( {\frac{r}{{r_m}}} \right)^2\left[ {\left( {\frac{{2r_m}}{{r + r_m}}} \right)^3 -\; \left( {\frac{{2r_m}}{{r_0 + r_m}}} \right)^3} \right] +\; \frac{{f^2r^2}}{4}} \right\}^{1/2} \,-\; \frac{{fr}}{2} \right\rangle}$$
  where *r*_*0*_ is the outer radius of the vortex beyond which *v* = 0. The quantities *v*_*m*_ and *r*_*m*_ are approximately the maximum wind speed and radius of maximum wind, respectively. Let the intensity decay linearly with height, so that *v* = 0 for *z* > *z*_sponge_. The vortex is a broad, weak, axisymmetric vortex^42^, which is placed in the middle of the domain. It is in hydrostatic and gradient–wind balance, with the maximum winds at the lowest model level. The relevant parameters of this specified vortex are listed in Eq. (6):
  6 $$\left\{ {\begin{array}{*{20}{c}} {r_0 = 412,500\;{\mathrm{m}}} \\ \hskip-0.5pc{r_m = 82,500\;{\mathrm{m}}} \\ \hskip-0.7pc{v_m = 15\;{\mathrm{m}}\;{\mathrm{s}}^{ - 1}} \\ {z_{{\mathrm{sponge}}} = 20,000\;{\mathrm{m}}} \end{array}} \right.$$
  Several relevant model parameters and parameterization schemes are shown in Supplementary Table 5, details of which can be found in the User Guide of the WRF model^43^.
  Jordan’s sounding is automatically meshed by the model at the beginning of the simulation. Since a vortex is added to the middle of the domain, as the model continues to integrate, the meshed sounding will control the evolution of the vortex. At the same time, under the influence of vortex changes, the sounding will also change over time.
- Experimental design
  Based on the description above, the default Jordan’s sounding and 28 °C SST in the WRF model is the CTRL experiment. According to the statistical results of ERA-Interim (Fig. 4b), we found that atmospheric stability has increased by about 2% in the last two decades. As the global surface temperature rises, the water vapor content in the atmosphere increases by about 5% (Fig. 4c), but the change of atmospheric water vapor is affected by SST in the model. Therefore, in order to simulate the effect of climate background changes on TC rainfall more closely, we increase the atmospheric stability by 2% in the exp_1 experiment based on Jordan’s sounding. Moreover, SST in exp_1 is increased by 0.5 °C to ensure that the moisture content in the atmosphere increases during the simulation.
  In order to increase atmospheric stability more realistically, the new potential temperature is defined as:
  7 $$\theta _{{\mathrm{exp}}\_1} = \theta _0 + (1 + 0.02) \times (\theta _{{\mathrm{CTRL}}} - \theta _0)$$
  where *θ*_exp_1_ and *θ*_CTRL_ in Eq. (7) are the potential temperatures on pressure level of exp_1 and CTRL experiments, and *θ*_0_ is the potential temperature of the bottom (sea surface level) in Jordan’s sounding.

## Supplementary information

Supplementary Information

## Data Availability

Best-track data of TCs are taken from IBTrACS-WMO v4 dataset (https://www.ncdc.noaa.gov/ibtracs/index.php?name=ib-v4-access). TC-related precipitation data are obtained from TMPA 3B42 v7 (https://disc.gsfc.nasa.gov/datasets/TRMM_3B42_V7/summary?keywords=TRMM_3B42), GPM IMERG Final Precipitation L3 Half Hourly 0.1 degree x 0.1 degree V06 (https://earthdata.nasa.gov/), ERA5 (https://cds.climate.copernicus.eu/cdsapp#!/home). The annual averaged TCHP is obtained from IAP Gridded temperature dataset (v3, 1 degree, monthly, 0–2000 m, http://159.226.119.60/cheng/). Total column water vapor and atmospheric stability are extracted from the monthly ERA-Interim dataset (https://apps.ecmwf.int/datasets/data/interim-full-moda/levtype=pl/). Source data has been uploaded with this paper. The processed data of this manuscript is available at 10.5281/zenodo.4533421. Source data are provided with this paper.

## Source data

Source Data

## Code availability

All of the codes used to read, calculate, and analyze the data are available at 10.5281/zenodo.4533421.

## References

[1] Jiang H, Zipser EJ (2010). Contribution of tropical cyclones to the global precipitation from eight seasons of TRMM data: regional, seasonal, and interannual variations. J. Clim..

[2] Lonfat M, Marks FD, Chen S (2004). Precipitation distribution in tropical cyclones using the Tropical Rainfall Measuring Mission (TRMM) microwave imager: a global perspective. Mon. Weath. Rev..

[3] Lin Y, Zhao M, Zhang M (2015). Tropical cyclone rainfall area controlled by relative sea surface temperature. Nat. Commun..

[4] Walsh KJE (2016). Tropical cyclones and climate change. Wiley Interdiscip. Rev. Clim. Change.

[5] Elsner JB, Kossin JP, Jagger TH (2008). The increasing intensity of the strongest tropical cyclones. Nature.

[6] Balaguru K, Foltz GR, Leung LR, Emanuel KA (2016). Global warming-induced upper-ocean freshening and the intensification of super typhoons. Nat. Commun..

[7] Sobel AH (2016). Human influence on tropical cyclone intensity. Science.

[8] Mendelsohn R, Emanuel K, Chonabayashi S, Bakkensen L (2012). The impact of climate change on global tropical cyclone damage. Nat. Clim. Change.

[9] Ren F, Wang Y, Wang X, Li W (2007). Estimating tropical cyclone precipitation from station observations. Adv. Atmos. Sci..

[10] Chang CP, Yang YT, Kuo HC (2013). Large increasing trend of tropical cyclone rainfall in Taiwan and the roles of terrain. J. Clim..

[11] Lau WKM, Zhou YP (2012). Observed recent trends in tropical cyclone rainfall over the north Atlantic and the north Pacific. J. Geophys. Res. Atmos..

[12] Knutson TR (2008). Simulated reduction in Atlantic hurricane frequency under twenty-first-century warming conditions. Nat. Geosci..

[13] Knutson TR (2010). Tropical cyclones and climate change. Nat. Geosci..

[14] Gualdi S, Scoccimarro E, Navarra A, Gualdi S (2008). Changes in tropical cyclone activity due to global warming in a general circulation model. J. Clim..

[15] Christensen, J. H. et al. in *Climate Change 2013: The Physical Science Basis. Contribution of Working Group I to the Fifth Assessment Report of the Intergovernmental Panel on Climate Change* (eds. Stocker, T. F. et al.) 1217–1308 (Cambridge Univ. Press, 2013).

[16] Kossin, J. P. et al. in *Climate Science Special Report: Fourth National Climate Assessment* Vol. I (eds. Wuebbles, D. J. et al.) 257–276 (US Global Change Research Program, 2017).

[17] Cheng, L., Abraham, J., Hausfather, Z., & Trenberth, K. E. How fast are the oceans warming? *Science*, **363**, 128–129 (2019). 10.1126/science.aav7619 30630919

[18] Sharmila S, Walsh KJE (2018). Recent poleward shift of tropical cyclone formation linked to Hadley cell expansion. Nat. Clim. Change.

[19] Allan RP, Soden BJ (2008). Atmospheric warming and the amplification of precipitation extremes. Science.

[20] Trenberth KE (2011). Changes in precipitation with climate change. Clim. Res.

[21] Rogers R., Marks F., & Marchok T. *Encyclopedia of Hydrological Sciences*. (Wiley Online Library, 2009).

[22] Bretherton CS, Peters ME, Back LE (2004). Relationships between water vapor path and precipitation over the tropical oceans. J. Clim..

[23] Ahmed F, Schumacher C (2015). Convective and stratiform components of the precipitation-moisture relationship. Geophys. Res. Lett..

[24] Liu P, Li C, Wang Y, Fu Y (2013). Climatic characteristics of convective and stratiform precipitation over the tropical and subtropical areas as derived from TRMM PR. Sci. China Earth Sci..

[25] Jordan CL (1958). Mean soundings for the West Indies area. J. Meteorol..

[26] Chen Y, Ebert EE, Walsh KJE, Davidson NE (2013). Evaluation of TMPA 3b42 daily precipitation estimates of tropical cyclone rainfall over Australia. J. Geophys. Res.: Atmos..

[27] Magnusson L (2019). Advances in understanding difficult cases of track forecasts. Trop. Cyclone Res. Rev..

[28] Chavas DR, Lin N, Emanuel K (2015). A model for the complete radial structure of the tropical cyclone wind field. Part I: comparison with observed structure. J. Atmos. Sci..

[29] Yang SK, Smith GL (1985). Further study on atmospheric lapse rate regimes. J. Atmos. Sci..

[30] Mokhov II, Akperov MG (2006). Tropospheric lapse rate and its relation to surface temperature from reanalysis data. Izvestiya, Atmos. Ocean. Phys..

[31] Knapp KR, Kruk MC, Levinson DH, Diamond HJ, Neumann CJ (2010). The international best track archive for climate stewardship (IBTrACS). Bull. Am. Meteor. Soc..

[32] Huffman GJ, Adler RF, Bolvin DT, Nelkin EJ (2007). The TRMM multi-satellite precipitation analysis (TMPA): Quasi-global, multiyear, combined-sensor precipitation estimates at fine scales. J. Hydrometeor.

[33] Huffman, G. J., Stocker, E. F., Bolvin, D. T., Nelkin, E. J., Tan, J. *GPM IMERG Final Precipitation L3 Half Hourly 0.1 Degree x 0.1 Degree v06, Greenbelt, MD, Goddard Earth Sciences Data and Information Services Center (GES DISC)* Accessed: July 2020, 10.5067/GPM/IMERG/3B-HH/06 (2019).

[34] Copernicus Climate Change Service (C3S). *ERA5: Fifth Generation of ECMWF Atmospheric Reanalyses of the Global Climate*. Copernicus Climate Change Service Climate Data Store (CDS), 24 Dec 2019. https://cds.climate.copernicus.eu/cdsapp#!/home (2017).

[35] Cheng L, Zhu J (2016). Benefits of CMIP5 multimodel ensemble in reconstructing historical ocean subsurface temperature variations. J. Clim..

[36] Dee D (2011). The ERA-Interim reanalysis: configuration and performance of the data assimilation system. Q. J. R. Meteorol. Soc..

[37] Harper BA, Kepert JD, Ginger JD (2010). Guidelines for Converting between Various Wind Averaging Periods in Tropical Cyclone Conditions.

[38] Leipper DF (1964). Observed ocean conditions and hurricane hilda. J. Atmos. Sci..

[39] Guo YP, Tan ZM (2018). Westward migration of tropical cyclone rapid-intensification over the Northwestern Pacific during short duration El Niño. Nat. Commun..

[40] Tu S (2020). Differences in the destructiveness of tropical cyclones over the western North Pacific between slow-and rapid-transforming El Niño years. Environ. Res. Lett..

[41] Skamarock, W. C. et al. A description of the advanced research WRF version 4. *NCAR Tech. Note NCAR/TN-556+STR*, 145 pp. 10.5065/1dfh-6p97 (2019).

[42] Rotunno R, Emanuel KA (1987). An air-sea interaction theory for tropical cyclones. Part II. Evolutionary study using a nonhydrostatic axisymmetric numerical model. J. Atmos. Sci..

[43] Wang, W. et al. In *User’s Guide for the Advanced Research WRF (ARW) Modeling System Version 4.1.* Available online: https://www2.mmm.ucar.edu/wrf/users/docs/user_guide_v4/v4.1/users_guide_chap5.html#examples (2019).

