# Supplementary material for: Recent global decrease in the inner-core rain rate of tropical cyclones
Source: Nat Commun. 2021 Mar 29;12:1948. doi: 10.1038/s41467-021-22304-y (PMC8007805; doi:10.1038/s41467-021-22304-y)
Supplement: Supplementary file 1 — Supplementary Information [file 41467_2021_22304_MOESM1_ESM.pdf]

# Supplementary Information

# Recent Global Decrease in the Inner-core Rain Rate of Tropical Cyclones

Shifei Tu[1], Jianjun Xu[1,2*], Johnny C L Chan[3], Kian Huang[1], Feng Xu[1], Long S Chiu[4]

1. South China Sea Institute of Marine Meteorology & College of Ocean and Meteorology, Guangdong Ocean University, Zhanjiang, China.
2. Shenzhen Institute of Guangdong Ocean University, Shenzhen, China.
3. School of Energy and Environment, City University of Hong Kong, Hong Kong, China.
4. Department of Atmospheric, Oceanic and Earth Sciences, George Mason University, Fairfax, Virginia, USA.

*. Corresponding author: E mail: jxu@gdou.edu.cn

**Supplementary Table 1 Details of all relevant datasets used in this work.**

| Datasets | Reference | Spatial resolution | Temporal resolution | Period | Sample size of TC rainfall |
|---|---|---|---|---|---|
| IBTrACS-WMO v4 | Ref. 1 | – | 3-hourly | 1999.01-2018.12 | – |
| TMPA 3B42 v7 | Ref. 2 | 0.25°×0.25° | 3-hourly | 1999.01-2018.12 | 49887 |
| GPM (IMERG Final Run) | Ref. 3 | 0.1°×0.1° | 0.5-hourly | 2000.06-2018.12 | 47475 |
| ERA5 | Ref. 4 | 0.25°×0.25° | 3-hourly | 1999.01-2018.12 | 49887 |
| IAP Gridded temperature dataset | Ref. 5 | 1°×1° | Monthly | 1999.01-2018.12 | – |
| ERA-Interim | Ref. 6 | 1°×1° | Monthly | 1999.01-2018.12 | – |

**Supplementary Table 2 Boundaries and the maximum gradient positions of tropical cyclone (TC) rainfall in six ocean basins with different TC intensities constructed by using TMPA dataset.**

| | Classification | WNP | ENP | NA | SIO | SP | NI | SA |
|---|---|---|---|---|---|---|---|---|
| Boundaries of TC rainfall | TSs | 525 | 325 | 400 | 450 | 525 | 450 | 125 |
| | CAT12 | 475 | 300 | 400 | 400 | 525 | 400 | 150 |
| | CAT35 | 500 | 350 | 400 | 425 | 525 | 400 | - |
| The maximum gradient position of TC rainfall | TSs | 125 | 125 | 125 | 125 | 125 | 125 | 125 |
| | CAT12 | 125 | 100 | 125 | 125 | 125 | 100 | 100 |
| | CAT35 | 125 | 100 | 125 | 125 | 100 | 100 | - |

WP: Western North Pacific, EP: Eastern North Pacific, NA: North Atlantic, SI: South Indian Ocean, SP: South Pacific, NI: North Indian Ocean. Unit: km. Note: there just has one TC generated over the SA (South Atlantic), with 21 effective instantaneous observations of rainfall during the period of 1999-2018.

**Supplementary Table 3 Changes (%) in the rain rate over the period 1999~2018 in various regions.**

| | Classification | Globe | NH | SH | WP | EP | NA | SI | SP | NI |
|---|---|---|---|---|---|---|---|---|---|---|
| All TC rainfall | TSs | **12±4** | **10±3** | **18±6** | **14±5** | 9±9 | 16±8 | **22±7** | 11±9 | 6±14 |
| | CAT12 | 5±4 | 7±5 | –4±7 | 8±5 | 11±11 | 8±9 | –5±8 | –6±9 | –21±28 |
| | CAT35 | –2±5 | –1±6 | –9±9 | 12±9 | 7±17 | 13±10 | –1±10 | –17±19 | 25±18 |
| | All | **8±4** | **8±4** | 8±6 | **14±5** | 8±9 | 12±8 | 10±6 | 4±8 | 9±13 |
| Inner–core | TSs | **–19±3** | **–19±4** | **–18±4** | **–21±5** | –13±6 | –11±6 | **–20±4** | –14±9 | –14±17 |
| | CAT12 | **–28±5** | **–27±6** | **–33±8** | **–29±7** | –13±8 | **–26±8** | **–35±9** | **–36±8** | –23±23 |
| | CAT35 | **–29±5** | **–29±6** | **–29±10** | –27±13 | **–23±11** | **–23±11** | **–37±9** | –29±25 | –8±30 |
| | All | **–24±3** | **–23±4** | **–26±5** | **–21±6** | –12±8 | **–22±9** | **–29±4** | **–22±8** | –11±16 |
| Outer region | TSs | **20±4** | **18±4** | **26±7** | **24±6** | **23±11** | **23±10** | **33±8** | 13±11 | 7±18 |
| | CAT12 | **14±5** | **16±5** | 4±9 | **17±6** | 26±15 | 18±12 | 4±9 | –3±11 | –25±31 |
| | CAT35 | 9±6 | 10±8 | 4±15 | 19±9 | 20±25 | 23±12 | 18±16 | –18±22 | 39±35 |
| | All | **17±4** | **17±4** | **18±7** | **23±5** | 21±11 | **21±9** | **24±7** | 6±10 | 12±17 |

Globe: Globally-average, NH: North Hemisphere, SH: South Hemisphere, WP: Western North Pacific, EP: Eastern North Pacific, NA: North Atlantic, SI: South Indian Ocean, SP: South Pacific, NI: North Indian Ocean. Percentages are obtained from the linear regression curve of rain rates. Bold is significant at the 95% confidence level ($P \leq 0.05$). The rain rates are constructed by using the Tropical Rainfall Measuring Mission (TRMM) Multi-Satellite Precipitation Analysis (TMPA) dataset.

**Supplementary Table 4 Correlation coefficients of atmospheric stability and total column water vapor with the rain rates of tropical cyclone.**

| Correlation | Inner-core | Outer region | Stratiform (Inner-core) | Stratiform (Outer region) | Stratiform (All) | Convective (Inner-core) | Convective (Outer region) | Convective (All) |
|---|---|---|---|---|---|---|---|---|
| AS | **–0.49** | **0.57** | 0.41 | **0.69** | **0.70** | **–0.50** | **–0.56** | **–0.55** |
| TCWV | **–0.73** | **0.73** | 0.19 | **0.83** | **0.82** | **–0.45** | –0.44 | **–0.48** |
| AS (excluding the influence from TCWV) | – | –0.03 | 0.26 | 0.04 | 0.08 | – | – | – |
| TCWV (excluding the influence from AS) | –0.39 | – | – | – | – | –0.05 | 0.01 | –0.05 |

AS: atmospheric stability, TCWV: total column water vapor. Bold is significant at the 95% confidence level (P ≤ 0.05).

**Supplementary Table 5 Relevant Model setting and parameterization schemes.**

| | Parameter | Value |
|---|---|---|
| Model Setting | Grid points | 300×300 |
| | Horizontal resolution | 12×12 km |
| | Vertical layer | 25 |
| | Model top | 25 km |
| | Coriolis parameter | $5.0\times10^{-5}$ $s^{-1}$ |
| | SST | 28.0 °C |
| | The length of integration | 360 hours |
| Physics Options (Ref. 7) | Microphysics | WRF Single–Moment 6–class scheme |
| | Longwave Radiation | Rapid Radiative Transfer Model for GCMs |
| | Shortwave Radiation | Rapid Radiative Transfer Model for GCMs |
| | Surface Layer | Yonsei University scheme |
| | Land Surface | Noah Land Surface Model |
| | Planetary Boundary layer | Yonsei University scheme |
| | Cumulus Parameterization | Tiedtke Scheme |

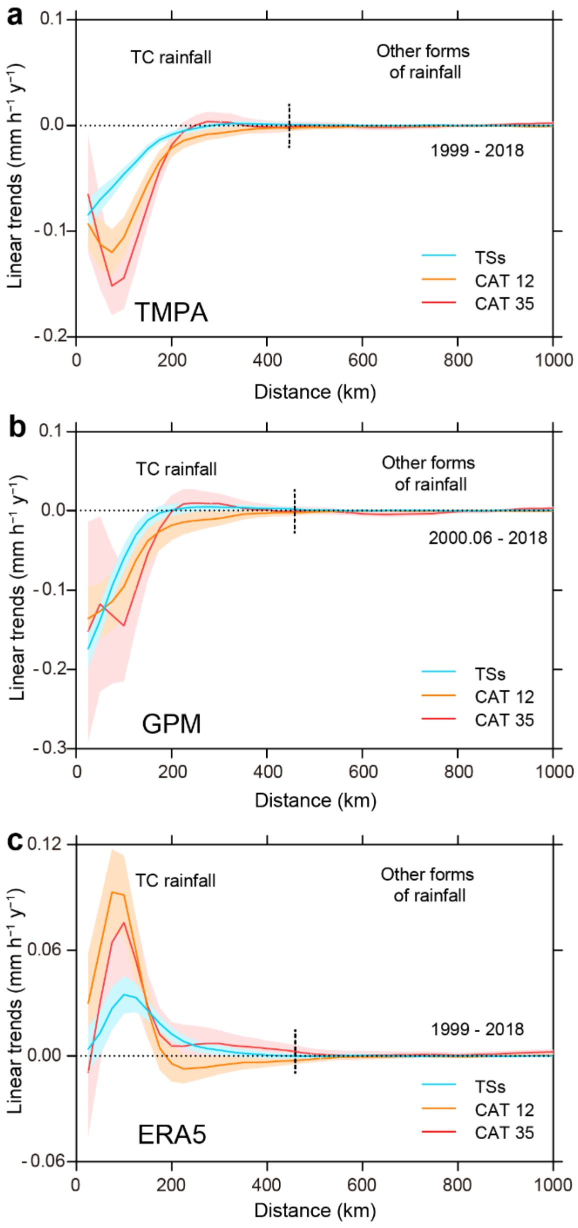


**Supplementary Figure 1 Radial distribution of linear trends of tropical cyclone (TC) rain rate based on different datasets, unit: mm h$^{-1}$ y$^{-1}$. a** the Tropical Rainfall Measuring Mission (TRMM) Multi-Satellite Precipitation Analysis (TMPA), **b** Global Precipitation Measurement (GPM), and **c** ERA5 dataset. Shaded areas indicate the standard error of linear trends of the TC rain rate. Blue: tropical storms (TSs), orange: categories 1-2 (CAT12), and red: categories 3-5 (CAT35). The vertical dotted line in each panel indicates the estimated boundary (~450 km for a global scale) of TC rainfall. All the linear trends here consider the area-average results (considering all pixels including rainy and non-rain).

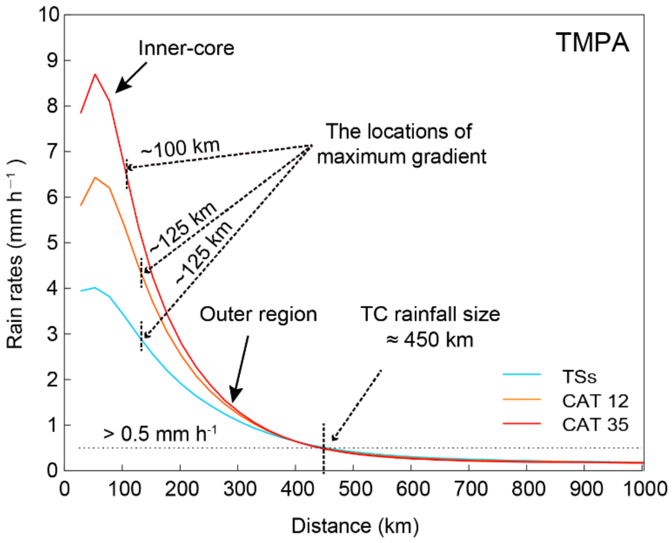


**Supplementary Figure 2 Schematic diagram of radial distribution of regional averaged tropical cyclone (TC) rain rate.** The colors are for different TC intensity categories; tropical storms (blue), category 1 and 2 (orange), and category 3 to 5 (red).

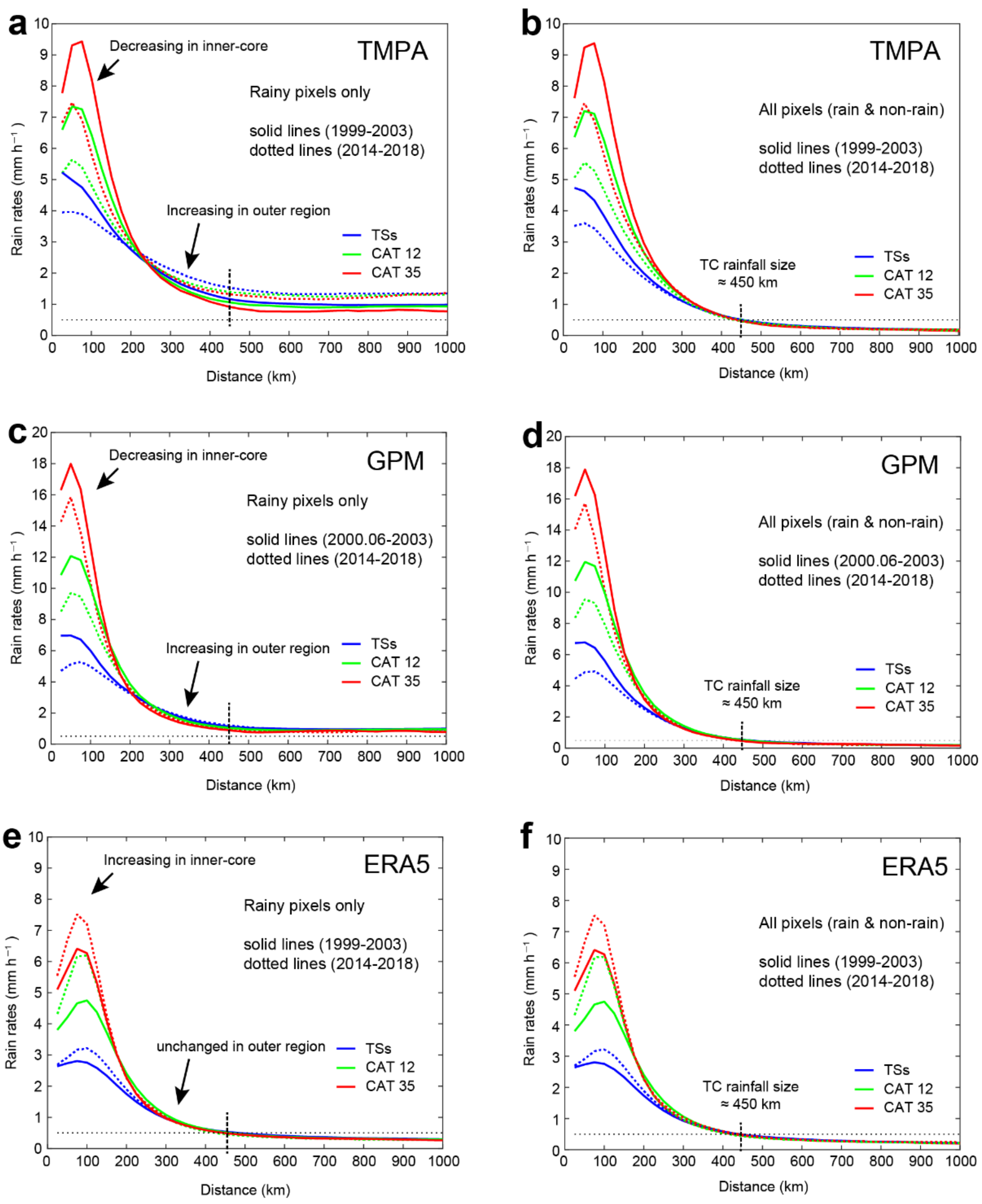


**Supplementary Figure 3 Radial distribution of rain rate (mm h$^{-1}$) of TC during the first and last five years. a** Rainy pixels only, **b** All pixels (including rainy and non-rain pixels) based on the Tropical Rainfall Measuring Mission (TRMM) Multi-Satellite Precipitation Analysis (TMPA). Solid lines indicated the average of first five years (1999~2003), and dotted lines indicated the average of the last five years (2014~2018). The colors are for different TC intensity categories; blue: tropical storms (TSs), orange: categories 1-2 (CAT12), and red: categories 3-5 (CAT35). **c**, **d**: Same as **a**, **b** except using Global Precipitation Measurement (GPM) data which have date between 2000.06 and 2018.12. **e**, **f**: Same as **a**, **b** except using ERA5 data.

## Supplementary References

1. Knapp, K. R., Kruk, M. C., Levinson, D. H., Diamond, H. J., & Neumann, C. J. The international best track archive for climate stewardship (IBTrACS). *Bull. Am. Meteor. Soc.* **91**, 363-376 (2010).
2. Huffman, G. J., Adler, R. F., Bolvin, D. T., & Nelkin, E. J. The TRMM multi-satellite precipitation analysis (TMPA): Quasi-global, multiyear, combined-sensor precipitation estimates at fine scales. *J. Hydrometeor*, **8**, 38-55 (2007).
3. Huffman, G.J., E.F. Stocker, D.T. Bolvin, E.J. Nelkin, Jackson Tan (2019), GPM IMERG final precipitation L3 half hourly 0.1 degree x 0.1 degree v06, greenbelt, MD, Goddard Earth Sciences Data and Information Services Center (GES DISC), Accessed: July 2020, https://doi.org/10.5067/GPM/IMERG/3B-HH/06
4. Copernicus Climate Change Service (C3S) (2017): ERA5: Fifth generation of ECMWF atmospheric reanalyses of the global climate. Copernicus Climate Change Service Climate Data Store (CDS), 24 Dec 2019. https://cds.climate.copernicus.eu/cdsapp#!/home
5. Cheng, L., & Zhu, J. Benefits of CMIP5 multimodel ensemble in reconstructing historical ocean subsurface temperature variations. *J. Clim.*, **29**, 5393-5416 (2016).
6. Dee, D. *et al*. The ERA-Interim reanalysis: Configuration and performance of the data assimilation system. *Q. J. R. Meteorol. Soc.* **137**, 553-597 (2011).
7. Chapter 5: WRF Model. Examples of namelists for various applications in *User's Guide for the Advanced Research WRF (ARW) Modeling System Version 4.1.* Available online: https://www2.mmm.ucar.edu/wrf/users/docs/user_guide_v4/v4.1/users_guide_chap5.html#examples. (2019)
